# Fluid responsiveness during weaning from mechanical ventilation

**DOI:** 10.1186/cc10843

**Published:** 2012-03-20

**Authors:** M Geisen, UM Schmid, O Dzemali, A Zollinger, CK Hofer

**Affiliations:** 1 Triemli City Hospital, Zürich, Switzerland

## Introduction

To overcome the limited accuracy of functional hemodynamic parameters such as stroke volume and pulse pressure variation (SVV and PPV) during spontaneous breathing, a passive leg raising (PLR) maneuver has been suggested as a reliable predictor of fluid responsiveness [1,2]. The aim of this study was to evaluate fluid responsiveness using SVV, PPV and PLR during the transition from controlled to spontaneous breathing.

## Methods

Thirty-four patients after off-pump CABG were enrolled. Measurements were performed in the ICU using a PiCCO_2 _system. Fluid (500 ml) was given: (A) during controlled mechanical ventilation, (B) during pressure support ventilation with spontaneous breathing and (C) after extubation. The stroke volume (SV), SVV and PPV as well as the mean arterial pressure and heart rate were assessed. A PLR was performed before fluid administration at all three time points. Fluid response was defined as an increase in SV >15%. Prediction of fluid responsiveness was tested using ROC analysis.

## Results

In 34 patients significant hemodynamic changes were observed, with 19 (55.9%), 22 (64.7%), and 13 (40.6%) responders at time points A, B and C, respectively. Prediction of fluid responsiveness is depicted in Table 1.

**Table 1:** Prediction of fluid responsiveness

	A			B			C		
			
	AUC	*P *value	TS%	AUC	*P *value	TS%	AUC	*P *value	TS%
SVV	0.88	0.0001	15.5	0.70	0.056	12.5	0.56	0.604	13.5
PPV	0.83	0.001	14.5	0.69	0.063	11.0	0.48	0.863	11.5
PLR SV%	0.72	0.028	8.0	0.74	0.021	10.0	0.70	0.058	8.0

TS, threshold.

## Conclusion

Prediction of a fluid response with SVV/PPV was less reliable in spontaneous breathing. PLR predicted fluid responsiveness, but was less accurate than previously reported.
